# Genetic comparison of transmissible gastroenteritis coronaviruses

**DOI:** 10.3389/fvets.2023.1146648

**Published:** 2023-04-17

**Authors:** Pei-Hua Wang, Amina Nawal Bahoussi, Pir Tariq Shah, Yan-Yan Guo, Changxin Wu, Li Xing

**Affiliations:** ^1^Institutes of Biomedical Sciences, Shanxi University, Taiyuan, China; ^2^Shanxi Provincial Key Laboratory of Medical Molecular Cell Biology, Shanxi University, Taiyuan, China; ^3^Shanxi Provincial Key Laboratory for Prevention and Treatment of Major Infectious Diseases, Taiyuan, China; ^4^The Key Laboratory of Chemical Biology and Molecular Engineering of Ministry of Education, Shanxi University, Taiyuan, China

**Keywords:** coronavirus, transmissible gastroenteritis virus (TGEV), porcine respiratory coronavirus (PRCV), phylogeographic network, phylogenetic tree, China, USA, recombination

## Abstract

Transmissible gastroenteritis virus (TGEV) is a porcine coronavirus that threatens animal health and remains elusive despite years of research efforts. The systematical analysis of all available full-length genomes of TGEVs (a total of 43) and porcine respiratory coronaviruses PRCVs (a total of 7) showed that TGEVs fell into two independent evolutionary phylogenetic clades, GI and GII. Viruses circulating in China (until 2021) clustered with the traditional or attenuated vaccine strains within the same evolutionary clades (GI). In contrast, viruses latterly isolated in the USA fell into GII clade. The viruses circulating in China have a lower similarity with that isolated latterly in the USA all through the viral genome. In addition, at least four potential genomic recombination events were identified, three of which occurred in GI clade and one in GII clade. TGEVs circulating in China are distinct from the viruses latterly isolated in the USA at either genomic nucleotide or antigenic levels. Genomic recombination serves as a factor driving the expansion of TGEV genomic diversity.

## Introduction

Porcine transmissible gastroenteritis virus (TGEV) is a member of the *Coronaviridae* family that was first identified in the United States of America (USA) in 1946 (1) and was reported subsequently in countries of Europe, Asia, Africa, and America (2). In China, TGEV infection was first detected in Guangdong province in 1956, and since then, sporadic outbreaks have been reported in multiple swine-producing provinces, including Heilongjiang, Jilin, Henan, Gansu, Shanghai, and Guangxi (3–7).

TGEV is a rapidly spreading pathogen that infects swine of all ages and species and causes enteritis with a mortality rate >90%, inversely correlated with the age of pigs (8–10). TGEV has a single-stranded positive-sense RNA genome of ~28.6 kb in length (11) and belongs to the subgenus *Tegacovirus* of the *Alphacoronavirus* genus (12). The viral genome encompasses at least nine open reading frames (ORFs). The 5' two-thirds of the virus genome contains ORF1a and ORF1b, encoding the polyprotein 1a (pp1a) and polyprotein 1b (pp1b), respectively, proteolytically processed by virus-encoded proteases into 16 nonstructural proteins (Nsps 1–16) during the translation (13). The 3' one-third of the virus genome encodes four viral structural proteins, including the spike glycoprotein (S), the envelope protein (E), the membrane protein (M), the nucleocapsid protein (N), and three accessory proteins (NS3A, NS3B, and NS7) (14).

The S glycoprotein of TGEV is a large type 1 membrane protein composed of 1448 amino acids (GenBank: KX499468.1, TGEV AHHF strain) (15) and containing five domains: N-terminal domain (NTD, aa 1–248) with a short peptide signal (aa 1–16) (16), S1 domain (aa 249–670), S2 domain (aa 832–1372) (15), the transmembrane domain (TM, aa 1388–1410), and C-terminal cytoplasmic tail (aa 1411–1448). S glycoprotein of coronaviruses is the main structural protein of the viral envelope, which mediates the entry of virions into the host cell and induces the generation of neutralizing antibodies (17). S glycoprotein of TGEV binds the sialic acid or the porcine aminopeptidase N (pAPN) to ensure the entry of virion into host cells (18). The cysteine-rich motif (CRM) at the TGEV S glycoprotein carboxy-terminus is palmitoylated, which facilitates the assembly of S glycoprotein during the maturation of viral particles (19). Mutational analysis of NTD of S glycoprotein revealed a mild effect on viral virulence (20–22).

A previous study of S glycoprotein of TGEV strain PUR46-MAD (GenBank: M94101.1; Protein ID: AAA47109.1) has identified four major epitope sites within the N-terminal first 543 amino acids in the order of C, B, D, and A (17). Sites A and B contain conformational epitopes that depend on glycosylation (23). The amino acid residues 538K, 591R, and 543G are essential to forming the antigen subsites Aa, Ab and Ac of site A, respectively. Site B also consists of at least three subsites, two of which are overlapped and include tryptophan. The amino acid residues 97W and 144S contribute to the formation of epitopes in site B. However, sites C and D contain glycosylation-independent linear epitopes located at aa 48–52 and aa 373–398, respectively (17, 24).

The non-enteropathogenic porcine respiratory coronavirus (PRCV) is a naturally occurring mutant of TGEV with the deletion of an N terminal segment of S glycoprotein (25). PRCV preferentially infects non-ciliated epithelial cells of the porcine respiratory tract (26).

Vaccination and immunoprophylaxis represent the most effective means to contain and prevent TGEV, in addition to biosecurity measures (27). Since coronaviruses are an ongoing threat to humans, animals and the global economy, this study aimed to review the evolution and molecular characteristics of the full-length genomes of TGEV and PRCV during past decades to help promote the control and surveillance strategies.

## TGEV and PRCV fall into two major genogroups GI and GII

**Figure 1:**
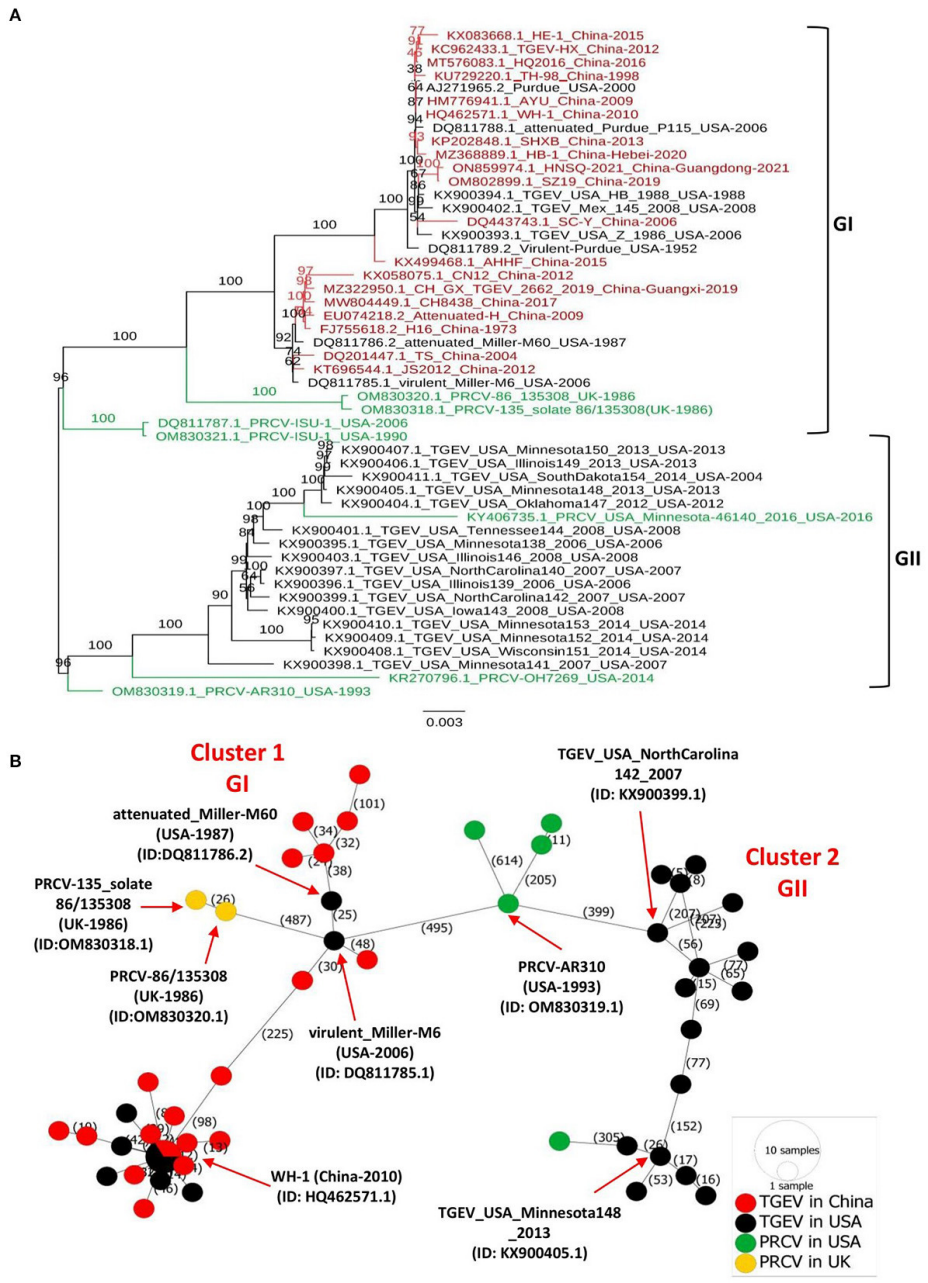
Phylogenetic tree and phylogeographic network of TGEV and PRCV strains. **(A)** Complete genome sequence-based ML tree. All complete genome nucleotide sequences were aligned using the MEGA11 software (37) and edited with the BioEdit v7.2.5. The ML phylogenetic trees were inferred using the IQ-TREE multicore version 1.6.12 (28) with 1000 bootstraps and the best-fitting model TIM + F + I + G4. The numbers on each branch are the bootstrap values (%). The scale bar represents a length corresponding to 0.003 nucleotide substitutions per site. Virus strains are formatted as GenBank accession number: virus name (country-year of collection). The red indicates TGEV from China, and the black indicates TGEV from the USA. The green color indicates PRCVs. **(B)** Phylogeographic network. The complete genome sequences of TGEV and PRCV were used to infer the Minimum Spanning Network (MSN) implemented by PopArt v1.7 (38). The network included 50 TGEV/PRCV strains, where 24 TGEV strains were from the USA, 19 TGEV strains from China, 5 PRCV strains from the USA and 2 PRCV strains from the UK. The mutation numbers are shown.

The full-length genomes of all available TGEVs, including 19 strains isolated in China between 1973–2021 and 24 strains isolated in the USA between 1952–2014, in addition to 7 PRCV viruses isolated in the USA and UK between 1986 to 2016 (Supplementary Table 1), were retrieved from the NCBI GenBank and aligned with the ClustalW using the MEGA11 software. The maximum likelihood (ML) phylogenetic trees based on the full-length genome sequences or genomic fragments containing different ORFs were inferred using the best-fitting models in the IQ-TREE multicore version 1.6.12 (28) with 1000 bootstraps. As shown in Figure 1A, the complete genome-based phylogenetic tree identified two main clades GI and GII. All viruses isolated in China fell into the GI clade, together with the eight earliest strains from the USA, including attenuated Purdue P115 (GenBank ID: DQ811788.1), Virulent Purdue (GenBank ID: DQ811789.2), virulent Miller-M6 (GenBank ID: DQ811785.1), attenuated Miller-M60 (GenBank ID: DQ811786.2), TGEV/USA/HB/1988 (GenBank ID: KX900394.1), TGEV/USA/Z/1986 (GenBank ID: KX900393.1), Purdue (GenBank ID: AJ271965.2), and TGEV/Mex/145/2008 (GenBank ID: KX900402.1). The remaining 16 viruses from the USA fell independently into a separate, distinct clade referred to as the GII clade (Figure 1A). To deeply understand the virus evolution, we further constructed phylogenetic trees based on different genomic fragments. In the phylogenetic trees based on ORF1a (Supplementary Figure 1A), and ORF1b (Supplementary Figure 1B), ORF2 (encoding spike glycoprotein of TGEV and PRCV) (Supplementary Figure 1C), and genomic fragment containing ORFs of E, M, and N proteins (Supplementary Figure 1D), all viruses isolated in China and the aforementioned relatively earliest or attenuated vaccine viruses identified in the USA clustered in GI clade, corroborating results of the full-length genome-based phylogenetic analysis (Figure 1). In addition, the phylogenetic tree based on the ORF2 (Supplementary Figure 1C) or genomic fragment containing ORFs of E, M, and N proteins (Supplementary Figure 1D) revealed that the PRCV strains, known as a natural mutant of TGEV with a segmental deletion in the ORF2, clustered independently according to their geographical regions. The two PRCV strains identified in the UK clustered into GI, whereas the five identified in the USA clustered genetically closer to each other into GII, within different sub-lineage (Figure 1, Supplementary Figures 1A, B), demonstrating a complex evolutionary relationship between TGEV and PRCV strains (29).

Before this report, all constructed phylogenetic trees based on the full-length genomes of 30 TGEV isolates (30), 23 isolates (8), or based on the ORF2 of 11 isolates (3), displayed two major clades. Herein, our study: (1) included all available full-length genomic sequences of TGEV and PRCV, (2) showed that the GII clade contains all viruses from the USA, whereas the GI clade encompasses the nine relatively old TGEVs or attenuated vaccine strains from the USA in addition to all TGEVs from China. These findings indicate that the genetic distance of TGEVs from China is relatively far from that of the strains from the USA in the GII clade.

To further determine the genetic relatedness of TGEV genomes, we conducted a genomic similarity analysis using SimPlot software (31) and involving nine representative TGEV isolates from each clade: GI clade (Supplementary Figures 2A, B) and GII clade (Supplementary Figures 2A, C), separately. The Virulent-Purdue TGEV (GenBank ID: DQ811789.2) was included as a query. The nucleotide sequences of China viruses revealed a great similarity (>97%) with the query strain for most parts of the viral genome (Supplementary Figures 2A, B). In contrast, the similarity between viral isolates of the GII clade and the query strain is significantly reduced to < 97% for most parts of the viral genome (Supplementary Figures 2A, C). Notably, the sequence similarity plot revealed that the ORF2 region shows the lowest similarity for viral isolates in both GI and GII clades (Supplementary Figures 2B, C, respectively). The genomic similarity plot results are consistent with the phylogenetic trees in that TGEVs isolated in China and USA belong to two distinct evolutionary branches. The low similarity of ORF2 region in the genome (Supplementary Figure 2) indicates that the spike glycoprotein is highly variable.

## Phylogeographic network of TGEV and PRCV

To map the regional spread and genetic relationships of TGEV and PRCV strains, we performed a phylogenetic network using all available complete genome sequences isolated from 1952 to 2021 (Supplementary Table 1). The analysis showed that strains within the GI and GII clades cluster into two separate Network Clusters (Figure 1B). The first Network Cluster (Cluster 1) consists of GI strains, while the second (Cluster 2) comprises GII strains (Figure 1B). These results support our ML phylogenetic classification of the complete genomes. Further, TGEV and PRCV strains seem to be radiated from strains isolated in the USA. The TGEV WH-1 (GenBank ID: HQ462571.1, China-2010) is identified sharing its genetic ancestor with the USA TGEV strains and connecting most of the GI strains to it through short mutational branches (Figure 1B). The remaining China TGEV strains are connected to the virulent_Miller-M6 (GenBank ID: DQ811785.1, USA-2006) within the same Network Cluster (Cluster 1). Interestingly, both the PRCV strains isolated in the UK, e.g., PRCV-86_135308 (GenBank ID: OM830320.1, UK-1986) and PRCV-135_solate 86/135308 (GenBank ID: OM830318.1, UK-1986) that were identified to be more distanced within GI clade of the full-length phylogenetic, are also connected to the virulent_Miller-M6 in the Network Cluster 1 (Figure 1B).

## Recombination of TGEV

Genomic recombination is a relatively common phenomenon for coronaviruses, which have also been reported to occur in TGEVs. Zhang et al. reported that the TGEV AHHF strain (GenBank: KX499468.1) is a natural recombinant (32). The ORF1a gene of AHHF resulted from the recombination between SC-Y (GenBank: DQ443743.1) and H16 (GenBank: FJ755618.2), while the spike gene of AHHF was produced by recombination between the virulent Purdue (GenBank: DQ811789.2) and the attenuated H strain (GenBank: EU074218.2). TGEV JS2012 (GenBank: KT696544.1) strain is another natural recombinant between Miller M6 (GenBank: DQ811785.1) and Purdue 115 (GenBank: DQ811788.1) (8). Thus, we performed further recombination analysis of the entire TGEV and PRCV genome sequences (from the USA and China) using the seven algorithms of the recombination detection program 4 (RDP, GENECONV, BootScan, MaxChi, Chimera, SiScan, and 3Seq) (33). The analysis identified the occurrence of at least four potential recombination events (Supplementary Table 2), three of which (Events 1–3) occurred within the GI clade, and one event (Event 4) occurred between strains in GII clade. The recombination among the USA strains (Event 4) is identified for the first time. In consistence with the previous reports (8, 32), the beginning and ending breakpoints of the identified recombination events are mainly located in ORF1a (Events 1 and 4) and ORF2-containg spike gene (Events 2 and 3) (Supplementary Figure 3). Zhang et al. report analyzed the occurrence of recombination among TGEV AHHF and 14 other strains and identified potential recombination at two locations (ORF1a and ORF2) (32). Consistent with those results, our analysis detected AHHF strain as a recombinant in two events (Events 1 and 2). In Event 2, the AHHF strain was recombined by CN12 and attenuated Purdue P115 in the ORF2, a new possibility of recombination for the AHHF virus. A previous report indicated that TGEV JS2012, a highly pathogenic strain isolated in newborn piglets faces in Jiangsu Province, China, resulted from natural recombination between Miller M6 and Purdue 115 and preserved the genetic integrity and characteristics of virulent strain, despite the recombination breakpoints in the ORF2 (8). Consistent with this report, TGEV JS2012 in our analysis was also identified as a recombinant strain, but between H16 (major parent) and the attenuated Purdue P115 (minor parent) in the ORF2, indicating the existence of alternative recombination. Interestingly, the identified recombination events occurred only between viruses in the same clade (intra-clade). None of the inter-clade recombinations have been found so far.

## The landscape of amino acid variation of the spike glycoprotein

**Figure 2:**
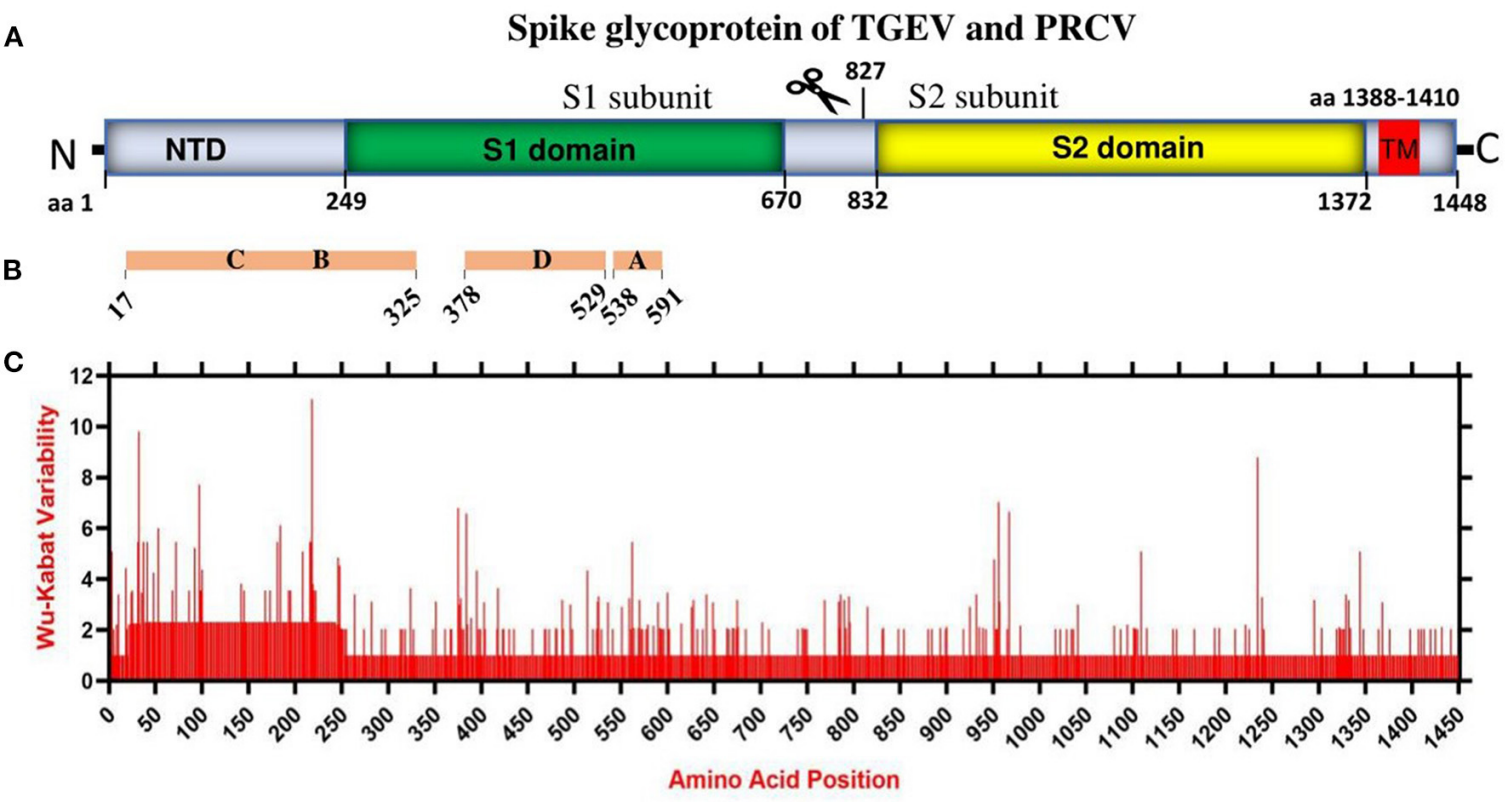
The amino acid variation landscape of TGEV and PRCV spike glycoprotein. **(A)** Domain structure of TGEV S glycoprotein, including NTD (aa 1–248), S1 domain (aa 249–670), S2 domain (aa 832–1,372), TM (aa 1,388–1,410) and C-terminal cytoplasmic tail (aa 1,411–1,448). **(B)** The brown blocks represent the relative positions of known sites containing neutralizing epitopes in the spike glycoprotein of TGEV (17). **(C)** Amino acid variation landscape. The amino acid sequences of all available complete spike proteins (a total of 58, Supplementary Table 1) of TGEV and PRCV were separately retrieved from the NCBI database and were aligned with ClustalW using the MEGA11 software (37). The protein variability was determined using the Wu-Kabat variability coefficient offered by the Protein Variability Server (PVS) (39). The Wu-Kabat variability coefficient describes the susceptibility of an amino acid position to evolutionary replacements and is computed using the following formula: variability coefficient = N*k/n, where N is the number of sequences in the alignment, k is the number of different amino acids at a given position and n is the frequency of the most common amino acid at that position. Y-axes represent the Wu-Kabat variability coefficient values, where the estimation limit is set as “1”. Above the limit of “>1” represents variations. X-axes represent the amino acid positions.

Coronaviruses enter the host cells by interaction between the S glycoprotein and the cellular receptor. S glycoprotein represents the chief immunogenic protein that elicits neutralizing antibody production (34) and the main antigen for vaccine research (35, 36). The virulence and antigenicity of TGEV S protein were shown to be sensitive to amino acid (aa) changes (3, 8). The mutation at aa 585 of TGEV HQ2016 engenders a serine-to-alanine change, affecting receptor binding and antigenicity (8). Here, the genome similarity and recombination analysis identified a low similarity of ORF2 (encoding spike glycoprotein) (Supplementary Figure 2) with potential recombination events (Supplementary Figure 3). In addition, the S1 subunit of TGEV spike glycoprotein has been shown to contain the neutralizing epitopes (17) (Figures 2A, B). Thus, we evaluated the amino acid variation patterns of TGEV and PRCV spike proteins by applying the Wu-Kabat variability coefficient provided by the Protein Variability Server (PVS). The method was used to acquire the consensus sequences of spike protein that consists of 1449 aa in TGEV (Figure 2C). The N terminal region, including both NTD and S1 domain, is the most variable, especially the aa positions 1–100, aa 200–250, aa 370–400, and aa 500–600, where the neutralizing epitopes have been identified (17).

Overall, China TGEV isolates are found to keep genetic features similar to that of older or attenuated vaccine strains so far, but distinct from that of the USA strains isolated latterly, which has implications in the monitoring, prevention, and control of TGEV. Furthermore, mutation is the modification of a gene resulted from insertion, deletion, or substitution of a single or multiple base units. Recombination occurs when the larger genetic fragments from at least two genomes are exchanged. The highly variable regions of spike proteins of TGEV and PRCV (Figure 2) and the recombination events (Supplementary Figure 3) demonstrate that both mutation and recombination are driving the expansion of genetic diversity and transmission of TGEV and PRCV, which need attention for the surveillance and vaccine development.

## Data availability statement

The original contributions presented in the study are included in the article/Supplementary material, further inquiries can be directed to the corresponding author.

## Author contributions

P-HW and LX conceived the study. P-HW, PT, and AN performed analysis. Y-YG provided resources in data analysis. P-HW and AN wrote the manuscript. CW and LX carried out administration. AN, PT, and LX revised the manuscript. All authors have read and approved the final manuscript.
