# Supplementary material for: Genetic comparison of transmissible gastroenteritis coronaviruses
Source: Front Vet Sci. 2023 Apr 17;10:1146648. doi: 10.3389/fvets.2023.1146648 (PMC10150923; doi:10.3389/fvets.2023.1146648)
Supplement: Supplementary file 1 [file Data_Sheet_1.PDF]

Supplementary Tables S1-2

Genetic Comparison of Transmissible Gastroenteritis Coronaviruses

Running title: Evolution of TGEV and PRCV

Pei-Hua Wang [1#], Amina Nawal Bahoussi [1#], Pir Tarqi Shah[1#], Yan-Yan Guo[1], Changxin Wu[1,2,3,4],

Li Xing[1,2,3,4]*

Supplementary Table S1. Information of the genomic sequences of TGEV and PRCV analyzed in this study. The full-length genomic sequences (1-51) were used to construct phylogenetic trees in Figure 1 and Supplementary Figure 1. For amino acid variation landscape analysis in Figure 2, both full-length genomic sequences (1-51) and partial genomic sequences (52-59) of TGEV and PRCV were used to deduce the amino acid sequences of spike glycoprotein.

No.	GenBank accession no.	Isolate	Collection year	Country	Clade*
1	KX499468.1	AHHF	2015	China	I
2	HQ462571.1	WH-1	2010	China	I
3	DQ811787.1	PRCV-ISU-1	2006	USA	I
4	KX900394.1	TGEV/USA/HB/1988	1988	USA	I
5	EU074218.2	Attenuated-H	2009	China	I
6	HM776941.1	AYU	2009	China	I
7	DQ443743.1	SC-Y	2006	China	I
8	DQ201447.1	TS	2004	China	I
9	KP202848.1	SHXB	2013	China	I
10	KX900402.1	TGEV/Mex/145/2008	2008	USA	I
11	ON859974.1	HNSQ-2021	2021	China	I
12	MZ322950.1	CH/GX/TGEV/2662/2019	2019	China	I
13	MW804449.1	CH8438	2017	China	I
14	OM830321.1	PRCV-ISU-1	1990	USA	I
15	OM830320.1	PRCV-86/135308	1986	UK	I
16	OM830318.1	PRCV-135_solate_86/135308	1986	UK	I
17	OM802899.1	SZ19	2019	China	I
18	MZ368889.1	HB-1	2020	China	I
19	MT576083.1	HQ2016	2016	China	I
20	KX900393.1	TGEV/USA/Z/1986(USA-2006)	2006	USA	I
21	KX083668.1	HE-1	2015	China	I
22	FJ755618.2	H16	1973	China	I
23	KT696544.1	JS2012	2012	China	I
24	KC962433.1	TGEV-HX	2012	China	I
25	KU729220.1	TH-98	1998	China	I
26	AJ271965.2	Purdue	2000	USA	I
27	KX058075.1	CN12	2012	China	I
28	DQ811788.1	attenuated_Purdue_P115	2006	USA	I
29	DQ811785.1	virulent_Miller-M6	2006	USA	I
30	DQ811789.2	Virulent-Purdue	1952	USA	I
31	DQ811786.2	attenuated_Miller-M60	1987	USA	I
32	KX900408.1	TGEV/USA/Wisconsin151/2014	2014	USA	II
33	KX900407.1	TGEV/USA/Minnesota150/2013	2013	USA	II

34	KX900406.1	TGEV/USA/Illinois149/2013	2013	USA	II
35	KX900405.1	TGEV/USA/Minnesota148/2013	2013	USA	II
36	KX900404.1	TGEV/USA/Oklahoma147/2012	2012	USA	II
37	KX900403.1	TGEV/USA/Illinois146/2008	2008	USA	II
38	KX900400.1	TGEV/USA/Iowa143/2008	2008	USA	II
39	KR270796.1	PRCV-OH7269	2014	USA	II
40	KY406735.1	PRCV/USA/Minnesota-46140/2016	2016	USA	II
41	KX900401.1	TGEV/USA/Tennessee144/2008	2008	USA	II
42	KX900399.1	TGEV/USA/NorthCarolina142/2007	2007	USA	II
43	KX900398.1	TGEV/USA/Minnesota141/2007	2007	USA	II
44	KX900397.1	TGEV/USA/NorthCarolina140/2007	2007	USA	II
45	KX900396.1	TGEV/USA/Illinois139/2006	2006	USA	II
46	OM830319.1	PRCV-AR310	1993	USA	II
47	KX900395.1	TGEV/USA/Minnesota138/2006	2006	USA	II
48	KX900411.1	TGEV/USA/SouthDakota154/2014	2004	USA	II
49	KX900410.1	TGEV/USA/Minnesota153/2014	2014	USA	II
50	KX900409.1	TGEV/USA/Minnesota152/2014	2014	USA	II
51	JQ693052.1	KT3	2012	South Korea	-
52	JQ693051.1	KT2	2012	South Korea	-
53	DQ001167.1	TSX	2005	China	-
54	JQ693050.1	DAE	2012	South Korea	-
55	JQ693049.1	133	2012	South Korea	-
56	AY587882.1	HN2002	2004	China	-
57	AY335549.1	TS	2003	China	-
58	Z24675.1	French isolate RM4	1993	France	-

* Full-length genome-based genotype classification.

Supplementary Table S2. Identification of potential recombination events in the genome of TGEVs isolated in China and the USA during 1952 - 2015. Seven algorithms (RDP, GENECONV, Bootscan, MaxChi, Chimaera, SiScan, and 3Seq) embedded in the RDP4 package (Martin, Murrell, Golden, Khoosal, & Muhire, 2015) were used to identify the potential recombination events.

**Event serial number**	**Recombinant**	**Major parent**	**Minor parent**	**Detection methods**						
	GenBank ID: Virus name (Country-Year)	GenBank ID: Virus name (Country-Year)	GenBank ID: Virus name (Country-Year)	R	G	B	M	C	S	T
1	KX499468.1:AHHF(China-2015)	DQ443743.1:SC-Y(China-2006)	FJ755618.2:H16(China-1973)	+	+	+	+	+	+	+
2	KX499468.1:AHHF(China-2015)	DQ811788.1:attenuated_Purdue _P115 (USA-2006)	KX058075.1:CN12(China-2012)	+	+	+	+	+	+	+
3	KT696544.1:JS2012(China-2012)	FJ755618.2:H16 (China-1973)	DQ811788.1:attenuated_ Purdue_P115(USA-2006)	+	+	−	+	+	+	+
4	*KX900401.1:TGEV/USA/ Tennessee144/2008(USA-2008)	KX900400.1:TGEV/USA/Iowa1 43/2008(USA-2008)	KX900405.1:TGEV/USA/ Minnesota 148/2013(USA-2013)	+	−	−	+	+	+	+

R, RDP; G, GENECONV; B, Bootscan; M, MaxChi; C, Chimaera; S, SiScan; and T, 3Seq; +, verified; -, not verified.

* The major or minor parent may be the actual recombinant due to the possibility of misidentification.

Reference

Martin, D. P., Murrell, B., Golden, M., Khoosal, A., & Muhire, B. (2015). RDP4: Detection and analysis of recombination patterns in virus genomes. *Virus Evol, 1*(1), vev003. doi:10.1093/ve/vev003
