# Supplementary material for: Genetic comparison of transmissible gastroenteritis coronaviruses
Source: Front Vet Sci. 2023 Apr 17;10:1146648. doi: 10.3389/fvets.2023.1146648 (PMC10150923; doi:10.3389/fvets.2023.1146648)

Supplementary Figures S1-3

Genetic Comparison of Transmissible Gastroenteritis Coronaviruses

Running title: Evolution of TGEV and PRCV

Pei-Hua Wang [1#], Amina Nawal Bahoussi [1#], Pir Tariq Shah[1#], Yan-Yan Guo[1], Changxin Wu[1,2,3,4], Li Xing[1,2,3,4]*

Supplementary Figure S1. The ML phylogenetic trees of TGEV and PRCV strains on the basis of ORF1a **(A)**, ORF1b **(B)**, ORF2 (encoding spike glycoprotein) **(C)**, and genomic fragment containing ORFs of E, M, and N proteins **(D)**. The nucleotide sequences were aligned using the MEGA11 software, and edited with the BioEdit v7.2.5. The ML phylogenetic trees were inferred using the IQ-TREE multicore version 1.6.12 with 1000 bootstraps and best-fitting models TIM+F+I for genomic fragment containing ORFs of E, M, and N proteins, TIM+F+G4 for ORF2 and ORF1a, while GTR+F+G4 for ORF1b. The trees were visualized and modified using the FigTree v1.4. The numbers on each branch are the bootstrap values (%). Scale bar represents a length corresponding to nucleotide substitutions per site. The red color indicates TGEV from China, and black color indicates TGEV from USA. The green color indicates PRCVs.

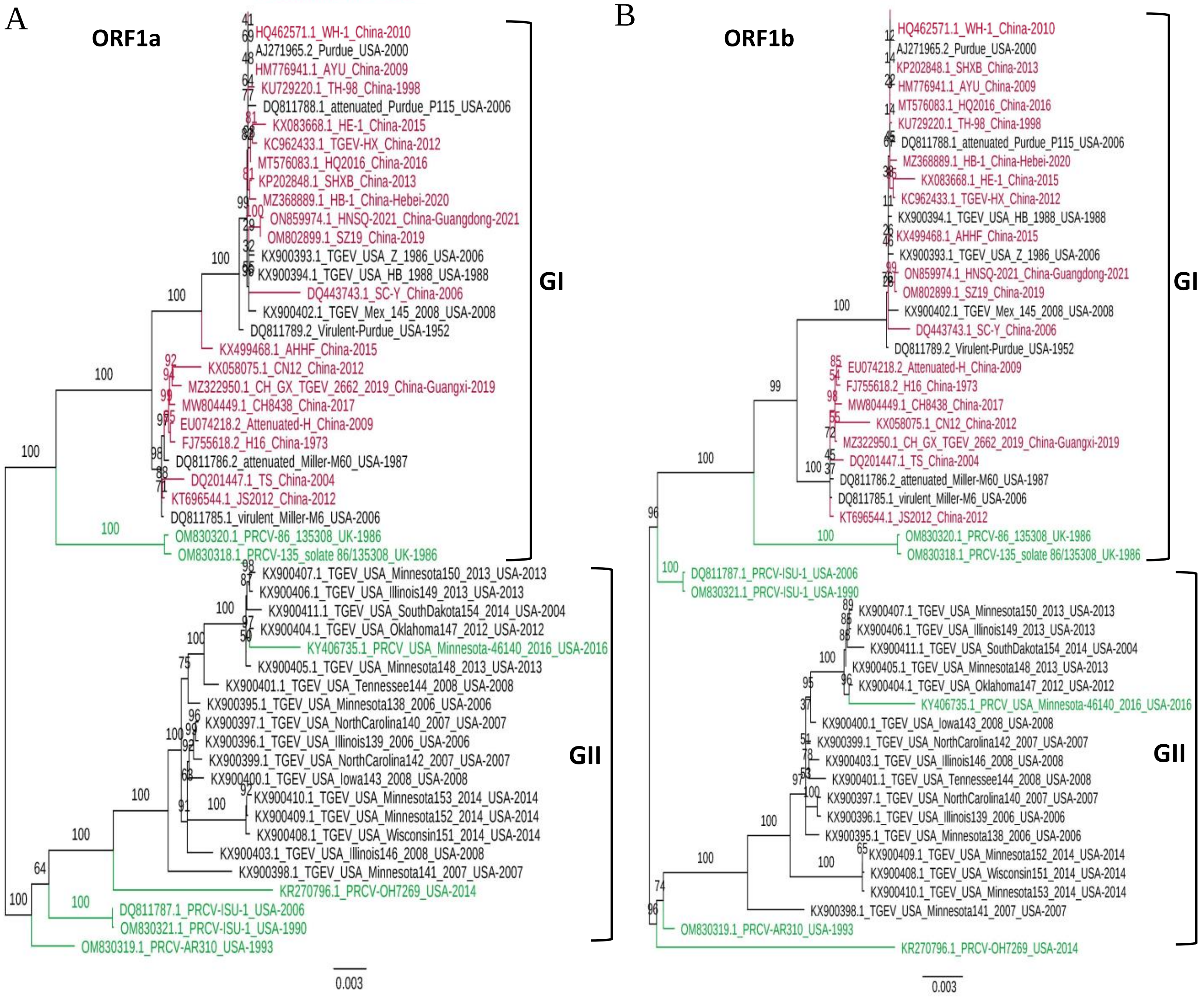

Supplementary Figure S1. Continued

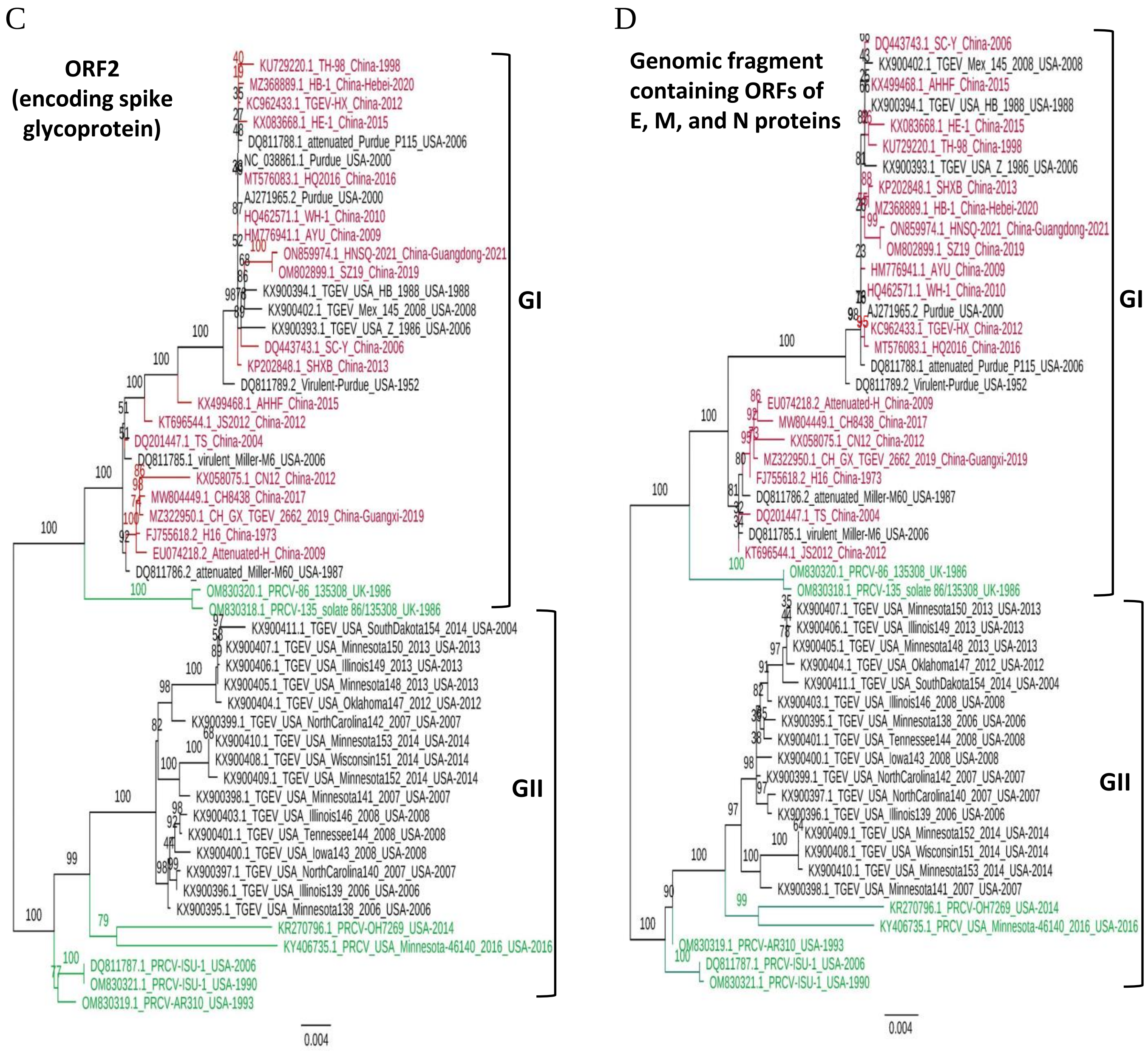

Supplementary Figure S2. Similarity analysis of full-length nucleotide sequence of TGEVs. **(A)** TGEV genome structure. Shown are ORF1a/1b, ORF2 (encoding spike glycoprotein), and ORFs encoding NS3a, NS3b, E, M, N, and NS7 proteins from 5' to 3' end of the viral genome. **(B)** Similarity plot of full-length nucleotide sequences of 9 isolates from GI clade with the query strain Virulent-Purdue/USA/1952 (GenBank ID: DQ811789.2). **(C)** Similarity plot of full-length nucleotide sequences of 9 representative isolates from GII clade with the query strain Virulent-Purdue/USA/1952. The query strain belongs to the GI clade. The vertical axes indicate the percentage of nucleotide similarity, and the horizontal axes indicate the nucleotide position (nt). Virus strains were represented with different colors.

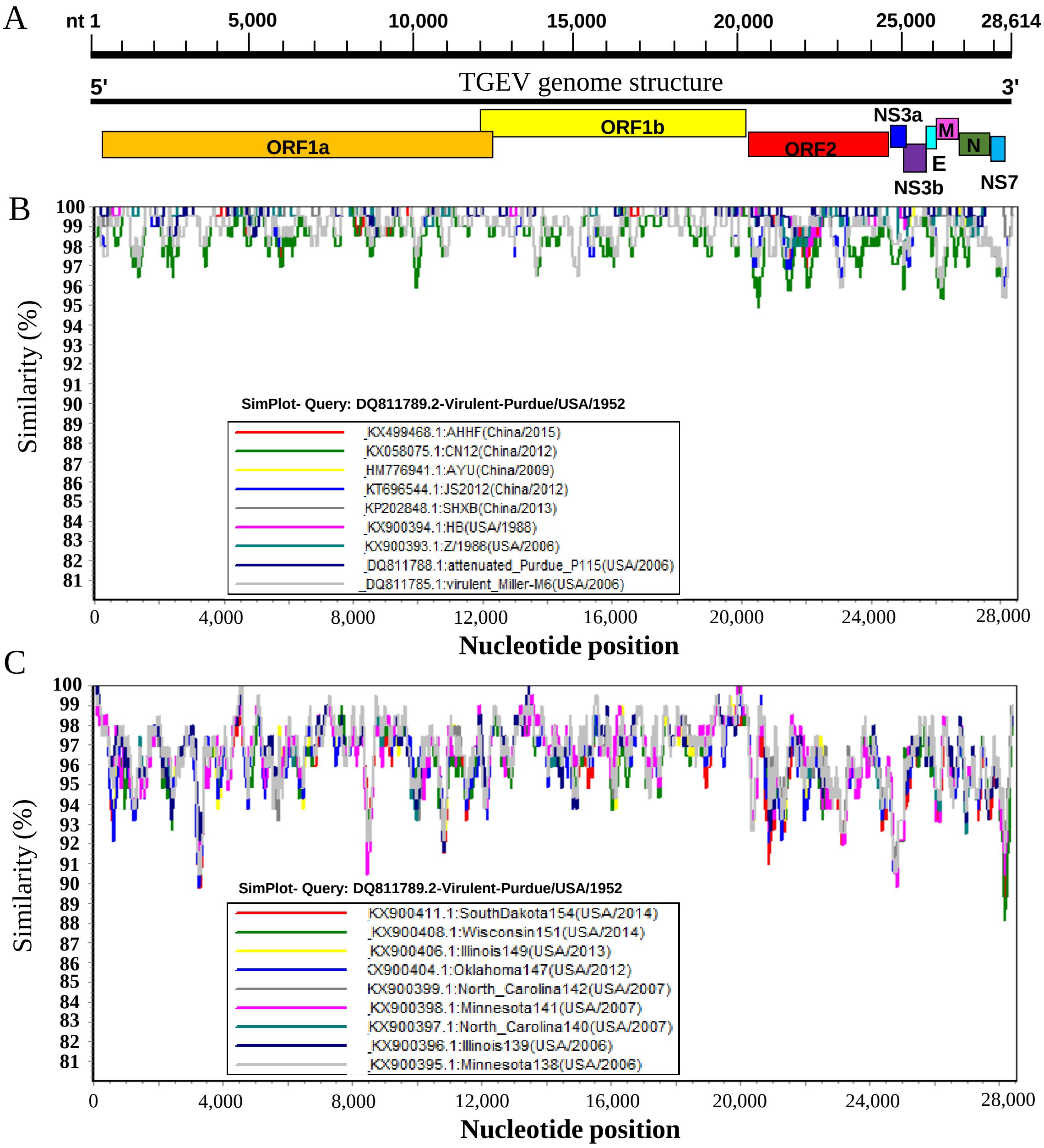

Supplementary Figure S3. Recombination analysis of TGEV/PRCV isolates. **(A)** The full-length genome structure of TGEV, including ORF1a/1b, ORF2 (encoding spike glycoprotein), and ORFs encoding NS3a, NS3b, E, M, N, and NS7 proteins from 5' to 3' end of the viral genome. . **(B)** Schematic diagrams showing four potential recombination events of TGEV isolates identified using the recombination detection program 4 (RDP4). The left side shows the serial numbers of the recombination events and the potential recombinants. The green block and pink block indicate the DNA region of the major parent viruses and minor parent viruses, respectively. The numbers indicate the positions of breakpoints relative to the full-length genome sequence of virus AHHF (GenBank ID: KX499468.1) identified in China in 2015.

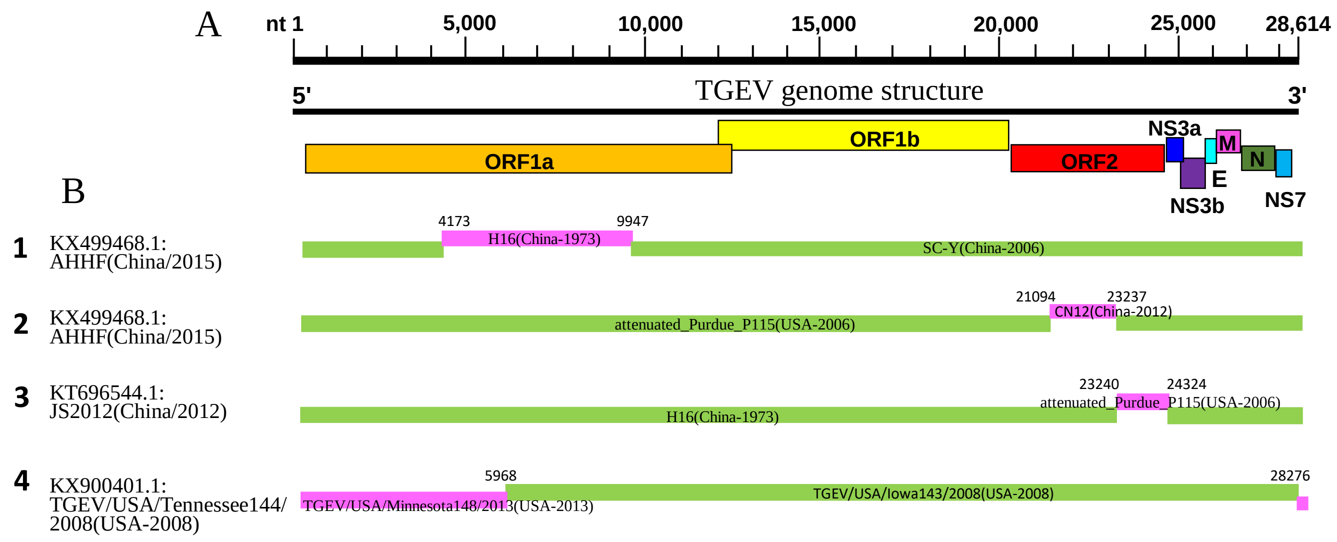

Supplement: Supplementary file 2 [file Data_Sheet_2.PDF]
